# Tackling reservoir siltation by controlled sediment flushing: Impact on downstream fauna and related management issues

**DOI:** 10.1371/journal.pone.0218822

**Published:** 2019-06-24

**Authors:** Paolo Espa, Ramon J. Batalla, Maria Laura Brignoli, Giuseppe Crosa, Gaetano Gentili, Silvia Quadroni

**Affiliations:** 1 Department of Science and High Technology, University of Insubria, Varese, Italy; 2 Fluvial Dynamics Research Group RIUS, University of Lleida, Lleida, Catalonia, Spain; 3 Catalan Institute for Water Research, ICRA, Girona, Catalonia, Spain; 4 Faculty of Forest Sciences and Natural Resources, Universidad Austral de Chile, Valdivia, Chile; 5 Pavia Acque S.c.a.r.l., Pavia, Italy; 6 Department of Theoretical and Applied Sciences, University of Insubria, Varese, Italy; 7 Graia S.r.l., Varano Borghi, Italy; Swedish University of Agricultural Science, SWEDEN

## Abstract

Sediment flushing can tackle reservoirs siltation and improve sediment flux through dammed rivers. However, the increase of the sediment loading below the dam can trigger a suite of undesired ecological effects in the downstream river reaches. To limit these drawbacks, sediment flushing can be controlled, by jointly regulating the sediment concentration of the evacuated water and the streamflow in the downstream channel. In this paper, we report on ten controlled sediment flushing operations (CSFOs), carried out between 2006 and 2012 in the central Italian Alps, at four hydropower reservoirs. These CSFOs displayed specific common traits: (i) Limits were set by the local environmental authorities concerning the allowable suspended sediment concentration. (ii) Reservoirs were fully drawn-down, earth-moving equipment was used to dislodge sediment, and the downstream water discharge was increased, compared to baseflow, by operating upstream intakes. (iii) Abiotic and biotic measurements in selected downstream reaches (before, during, and after the CSFOs) represented an integral part of the operations. In contrast, significant differences characterize the hydropower facilities (elevation and storage of reservoirs, in particular) as well as the basic CSFOs parameters (i.e., season, duration, mass and grain-size of the evacuated sediment, suspended sediment concentration). The macroinvertebrate assemblages resulted noticeably impacted by the CSFOs. In the short term, a significant density drop was observed, slightly influenced by the extent of the perturbation. In contrast, the latter appeared to control the assemblages contraction in terms of richness, according to the different sensitivity to sediment stress of the different taxa. The time employed to recover pre-CSFO standard ranged from few months to just under one year, and the related patterns would seem mostly correlated to the flushing season and to further site specificities. The density of trout populations was impacted as well, thus suggesting the adoption of mitigating strategies as removal by electrofishing before, and repopulation after the CSFO.

## Introduction

Reservoirs siltation and related issues are receiving growing attention due to the aging of water-storage infrastructures, particularly in North America and Europe, where most of the dam building took place during the 1940-1970s [1–6]. At the same time, the already mature knowledge of hydrological and geomorphological alteration due to river damming [7–8], coupled to the increasing expectancy of environmental improvement, are triggering the demand for managed sediment regimes in river systems characterized by flow regulation [9–12].

Sediment flushing can support tackling of the storage lost due to siltation, preserving at the same time the downstream sediment flux through dams, at least the small-sized fractions [13–14]. The need to manage reservoir siltation, dealing with sediment-laden water, achieves perhaps the most notable examples in China, where sediment flushing during high-flows is commonly performed to counteract the loss of storage in large multi-purpose reservoirs. For instance, the well-known practice of “storing clear water and releasing muddy water” is the basis of the management of the Sanmenxia Reservoir on the Yellow River since 1973, as well as of the planning and the current operation of the Three Gorges project on the Yangtze River [15–17]. Furthermore, flushing sediments through reservoirs is recognized to be of crucial importance for catchments such as the Mekong, where hydropower development is taking place at high pace, but where the massive sediment load sustains biodiversity and landforms of global importance [18]. The mitigation of the hydropower impacts on river ecology and hydro-morphology, including reservoir siltation and downstream sediment starvation due to trapping behind dam structures, is currently recognized as a priority issue also in the European Alps [19], where hydroelectricity of strategic importance at the regional and continental scales is generated long since [20].

However, sediment flushing operations can be highly detrimental for downstream river reaches, both in terms of siltation of instream-structures and from an ecological point of view [15]. Consequently, in the last years, the research concerning sediment flushing has shifted the focus from the old perspective of maximizing the flushing efficiency, i.e., maximizing the volume of flushed sediment per fixed volume of water [21–22], to up-grading flushing schedule and implementation in order to reduce the downstream ecological impacts [23–24].

Several quantitative parameters characterize a flushing event, and therefore influence its environmental impact in the downstream river reaches. These variables include:

- The evacuated sediment volume,
- The water amount allocated to transport and dilute the sediment below the flushed reservoir (including possible mitigation releases implemented after the flushing),
- The duration of the event,
- The sediment concentration of the flushed waters, in terms of both averaged and peak values,
- The streamflow, in terms of both averaged and peak values,
- The grain-size of the evacuated sediment, mostly controlling downstream transport and deposition.

Most of these variables are strictly interlocked, as partly discussed in the Supplementary Information (S1 File). For instance, when the evacuated sediment volume is fixed, the allocated water amount determines the sediment concentration of the flushed waters, and so on. However, at least to a certain degree, these variables can be managed in regulated systems, thus modulating the downstream impact. Obviously, this management includes trading-off with a set of technical/economical constraints, essentially related to the water use and its value, and to the actual possibility of regulation provided by the specific hydraulic scheme under consideration. Anyway, when the sediment flushing also includes objectives of downstream environmental safeguard, it can be defined as “controlled”, and we adopt here and throughout the text the acronym CSFO to indicate a Controlled Sediment Flushing Operation.

With the notable exceptions that will be recalled in the following, CSFOs are poorly discussed in the literature: this can be attributed to the relative novelty of the practice, and to the certainly great effort connected to both operatively control sediment flushing through reservoirs and properly survey CSFOs and assess their effects on the river environment. Consequently, the research on CSFOs is actually open to multidisciplinary up-to-date scientific contributions [6].

Within this context, we here examine ten CSFOs carried out between 2006 and 2012 at four hydropower reservoirs, located in the Central Italian Alps. As in the entire Alpine Region, hydropower is massively developed across the Italian Alps (51,000 km^2^, corresponding to 27% of the Alpine Region in terms of area). There, 210 large reservoirs (e.g., storage > 1 Mm^3^ or dam height > 15 m) can store approximately 2.2 km^3^ of water, mainly for hydropower purpose [19]. Moreover, 170 major units (i.e., installed power > 10 MW) result in 14.4 GW cumulated power (31% of the corresponding power installed in the Alpine Region), complemented by over 1,300 smaller units [25]. The mentioned CSFOs were planned and implemented to keep their downstream impact at a level considered acceptable. Field monitoring before, during, and after the events represented a basic support and a peculiar trait of the reported CSFOs. In this paper, we summarize and, when possible, generalize our main findings, also suggesting possible research development as emerging by our “pioneering” experience on the subject. Specifically, the main objectives of this work are: i) To concisely review the mentioned CSFOs, focusing on the main related issues and the quantitative parameters outlined above, and ii) To comparatively analyze the effects of these sediment releases on the main biophysical elements in the downstream river sections, with special emphasis on fish and benthic macroinvertebrates. In doing this, we tested some basic hypotheses, essentially related to the expected proportionality between the extent of the physical disturbance (primarily sediment concentration and duration of the CSFO) and the resulting impact on the aquatic fauna (e.g., decrease of fish density, particularly the younger specimens, and decrease of density and richness of the benthic macroinvertebrate assemblages).

## Materials and methods

### Study area and hydropower projects

**Fig 1:**
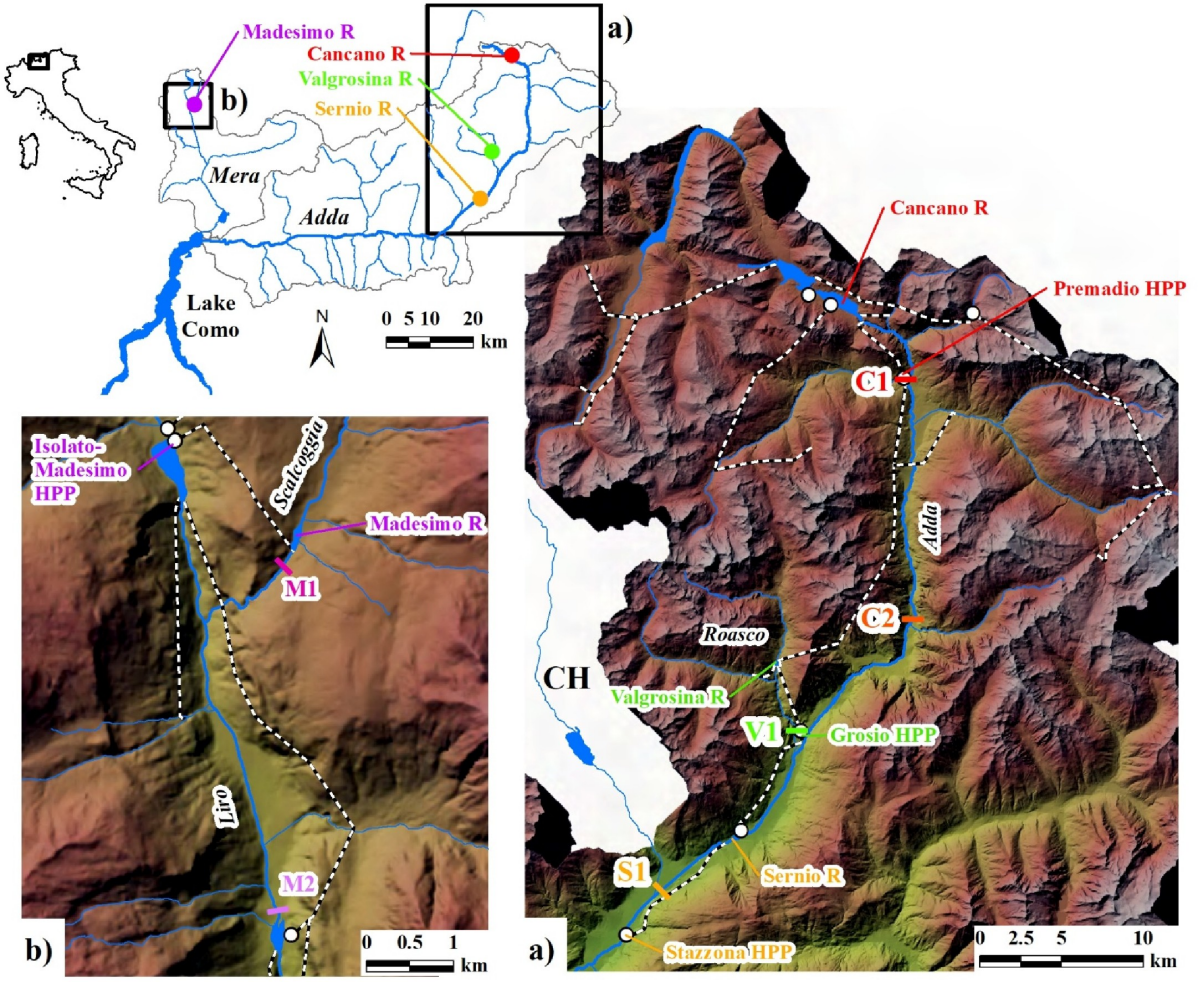
Location map. Colored circles indicate the four flushed reservoirs. Monitoring reaches (colored lines) and hydropower facilities (dashed lines indicate the main water-ducts, white circles indicate hydropower plants—HPPs) are reported in the Upper Adda River basin (a) and in the Liro River basin (b). Here and in the next figures, we adopted different colors to differentiate reservoirs and related measurements: red (CR), green (VR), yellow (SR), and purple (MR).

The CSFOs concerned four hydropower-reservoirs in the Central Italian Alps (Fig 1):

- Cancano Reservoir (hereafter CR), where three CSFOs took place in 2010, 2011, and 2012;
- Valgrosina Reservoir (hereafter VR), where four CSFOs were carried out from 2006 to 2009, once per year;
- Sernio Reservoir (hereafter SR), where two CSFOs took place in 2009 and 2010;
- Madesimo Reservoir (hereafter MR), where one CSFO was performed in 2010.

Reservoirs and related hydropower plants (Table 1) are located in the Lake Como catchment, in the north of Italy. Specifically, CR, VR, and SR are in the catchment of the Adda River (2,600 km^2^), the main tributary of Lake Como. MR is in the catchment of the Mera River (760 km^2^), the second main tributary of the lake. Both catchments are transboundary, with Swiss quotas of approximately 18.5% and 13% in terms of basin area, for Adda and Mera, respectively. They are massively developed for hydropower, with overall installed power exceeding 2.4 GW, and average annual productivity of approximately 6 TWh, thus resulting in a peculiarly high intensity of the hydropower sector, as assessed by Quadroni et al. [20].

**Table 1:** Main characteristics of the four flushed reservoirs and of the supplied hydropower plants (HPPs).

**Reservoir** **ID**	**Cancano** **CR**	**Valgrosina** **VR**	**Sernio** **SR**	**Madesimo** **MR**
**CAP** (Mm^3^)	124	1.3	0.7	0.13
**Z**_**TOP**_ (m_ASL_)	1,901	1,210	497	1,524
**A**_**TOT**_^**a**^ (km^2^)	370	712	885	25
**MAR** (Mm^3^)	271	533	757	41
**HPP**	**Premadio**	**Grosio**	**Stazzona**	**Isolato-Madesimo**
**IC** (MW)	226	430	30	17.3
**EH** (m)	647	600	88.7	273.5
**Q**_**MAX**_ (m^3^ s^-1^)	40	80	40	8

CAP = capacity, Z_TOP_ = elevation of the top of the active pool, A_TOT_ = total catchment area at the dam section, MAR = mean annual runoff, IC = installed capacity, EH = effective head, Q_MAX_ = maximum power station discharge.

^a^ A_TOT_ corresponds to the natural catchment area (A_NAT_) for SR and MR; A_NAT_ is 5.4% and 8.4% of A_TOT_ for CR and VR, respectively.

CR, VR, and SR belong to the same hydropower complex developing the upper basin of the Adda River and mostly providing peak energy (Fig 1A and S1A Fig). This system is dominated by two large contiguous reservoirs, San Giacomo and Cancano, with overall storage of 188 Mm^3^. Most of the upper Adda catchment above 2,000 m a.s.l. is channeled into the mentioned reservoirs, including the Spöl sub-catchment, which naturally drains into the Inn-Danube basin. After a first main jump, most of the same catchment above 1,200 m a.s.l. is conveyed into the VR, supplying the larger hydropower plant of the area (Table 1). Two further downstream hydropower plants complete the cascade.

MR is a small reservoir, belonging to the hydropower system developing the Liro River catchment (Fig 1B and S1B Fig): the Liro is the main tributary of the Mera River. MR is located on a tributary of the Liro River (i.e., the Scalcoggia Stream), and supplies a peak-energy plant discharging into a further reservoir located on the Liro River.

The mentioned catchments develop in mountainous areas (maximum elevations of 3,700 and 3,275 m a.s.l., for Adda and Liro catchments, respectively). The basins are scarcely anthropized, with prevailing activities (except for hydropower) mostly concentrated in the valley bottoms, and mostly related to mountain tourism. Sites of high naturalistic value are also present, including the Stelvio National Park in the upper Adda catchment. The average annual precipitations in the Adda and Mera catchments are 1,100 and 1,700 mm, respectively. The corresponding water-yields at the dam sections considered in the study are provided in Table 1. As expected for Alpine highlands, maximum seasonal runoff occurs between late spring and early summer, due to snow melting. Cold and well-oxygenated water flows in the rivers of the area, with generally low suspended sediment content, thus providing suitable habitat for salmonid fish. According to water-quality analyses routinely performed by the local environmental protection agency in the investigated watercourses, suspended sediment concentration (SSC) at baseflow is usually lower than 10 mg L^-1^, and dissolved oxygen (DO) higher than 10 mg L^-1^. Lastly, according to modeling data provided by the European Environment Agency [26], comparable erosion rates can be estimated characterizing the catchments of the Mera River closed at Lake Como and of the Adda River closed at SR, e.g., around 10–15 tons per hectare per year.

### Planning and implementation of the CSFOs

The CSFOs were planned and implemented accounting for specific restrictions set by the local environmental authorities. Specifically, the duration (ED) of the CSFO and the SSC averaged over ED (hereafter SSC_AVE_) were constrained to limit the fish mortality. The latter was estimated according to the dose-response model by Newcombe and Jensen [27]. Specifically, this model provides the fish response to increasing fine sediment in the water column through the severity of ill effect (SEV according to authors), an index ranging from 0 (no behavioral effect) to 14 (80–100% mortality). SEV is a simple function of SSC_AVE_ and ED, plus three regression coefficients provided by the authors according to fish species and life stage (see equation 1 in S1 File). As brown trout (*Salmo trutta fario* L.) is the dominant fish species in the study area, the coefficients estimated for salmonids (all ages) were adopted. It is worth to remark that the sediment considered by the mentioned model has grain size in the range 0.5–250 μm (i.e., clay to fine sand).

Even if the searching for optimal solutions was a rather difficult trial and error process, involving numerous river stakeholders, its essential rationale can be summarized as follows (further detail is provided in S1 File):

- Adoption of a suitable SEV value;
- Adoption of a suitable ED value;
- Determination of the corresponding SSC_AVE_ by equation 1 in S1 File;
- Evaluation of the evacuated volume of sediment and of the required water volumes in light of expected water availability and of the actual possibility of managing the system.

In general, the adopted SEVs were around 10 (corresponding to 0–20% fish mortality, increased predation and moderate to severe habitat degradation), except for the CSFOs at CR, no doubt the more complex works (SEV = 11; 20–40% mortality) [28] (Table 2). The wide uncertainties connected to the adoption of the model by Newcombe and Jensen [27] as a predictive tool induced to require fish monitoring before and after the CSFOs.

**Table 2:** CSFO parameters for the four flushed reservoirs at planning stage.

Reservoir ID	Site ID	SEV	Period	ED (days)	SSC_AVE_* (g/l)
**CR**	**C2**	11	Feb-Apr	40–50	3
**VR**	**V1**	10	Aug-Sep	12–13	4
**SR**	**S1**	10	May-Jul	15^a^	1.5
**MR**	**M2**	10	Oct	3	10

SEV = severity of ill effect according to Newcombe and Jensen [27], ED = duration of the event, and SSC_AVE_* = suspended sediment concentration (SSC) averaged over ED calculated using the Newcombe and Jensen [27] formula for salmonids—all ages (see equation 1 in S1 File) and permitted at monitoring reaches downstream of the flushed reservoir (see Fig 1).

^a^ non-consecutive days

A further constraint set by the local environmental authorities concerned the stream quality as assessed through the current Italian normative index based on benthic macroinvertebrates: the stream quality would have regained pre-flushing standard within periods not much longer than approximately six months. Consequently, benthos monitoring was required as well, to quantify the CSFO impact and recovery times.

The level of uncertainty related to the downstream impact of the CSFOs was even more prominent for the chronologically first ones: the CSFOs at VR in 2006 and 2007 had therefore a peculiar character of pilot case studies [29].

Control of the downstream SSC to comply with the required standards was carried out in similar ways in the reported case studies:

- Reservoirs were fully drawn-down (i.e., empty flushing);
- Mechanical equipment was employed during daytime to dislodge sediment and to move it into the flushing channel (S2 Fig);
- The water inflow in the emptied reservoir and the streamflow in the downstream reaches were regulated by operating upstream intakes and reservoirs;
- SSC was continuously gauged by one-point probes, using the raw data to possibly re-design the on-going activities, in case of recording unexpected values.

Each one of the previously listed points would require extensive discussion, concerning the detail of the adopted decisions and the related difficulties met in their practical implementation. Some essential remarks can be summarized as follows:

- Full draw-down was extremely difficult at CR [28] and, to a minor extent, at VR: in both cases, the small bottom-outlet facilities were fully submerged by thick layers of silt. As a consequence, the control of the downstream SSC was lost during the final emptying of these reservoirs through their bottom outlets, and undesired SSC peaks occurred.
- The use of earth-moving equipment was effective in removing sediment deposits, offering the possibility to focus the dislodging works on more sensitive reservoir areas, e.g., close to the power intakes. Moreover, regulating the pace of the work by mechanical equipment enabled a satisfactory control of the downstream SSC.
- Regulating the water inflow in the emptied reservoir and the streamflow in the downstream reaches was as much as important to control the downstream SSC. For instance, the water inflow into VR was interrupted during draw-down to avoid extremely high SSC at that moment (S2 Fig). Conversely, the CSFOs at SR were conducted with large inflow, fully operating upstream hydropower plants [30]. Anyway, to increase sediment dilution and transport capacity, the streamflow in the downstream reaches was always increased during the CSFOs compared to baseflow (S3 Fig).
- Further actions to keep the downstream SSC under control included an instream settling basin and off-stream diversion in case of very high SSC, but they were implemented only in the CSFOs at CR [28].
- The SSC measurements by optical turbidimeters were used for real-time monitoring and control as specified above. However, proper calibration of the probes output could be performed only when the CSFOs were completed, i.e., after lab analysis of the turbid water samples collected for this purpose.

The selection of the proper time of the year for executing CSFOs was also a key aspect of the discussed projects. In general, efforts were made to avoid winter (Table 2), i.e., the most sensitive period for trout, due to spawning and fry emergence. Again, the CSFOs at CR represented a notable exception: there, low winter temperatures and flows gave the only possible technical alternative to perform a CSFO, due to the large capacity of the reservoir and its small bottom outlet [28]. The season of maximum runoff was also discarded for the CSFOs at VR and MR, basically to improve the control capabilities of the systems and the safety conditions for the employment of earth-moving equipment in the dried reservoirs. Only at SR, it was feasible to perform the CSFOs during maximum seasonal runoff; moreover, to further decrease the downstream impact, CSFOs at SR were implemented along non-consecutive days [30].

### Monitoring sites

Selection of the downstream monitoring reaches also represented a key aspect of the CSFO projects. An effort was generally made to survey more than one downstream reach per CSFO. The aim was to quantify the gradient of the downstream perturbation and the related gradient in terms of impact on the biota and subsequent recovery. In fact, both perturbation and impact were expected to decrease in downstream direction as a result of deposition and dilution due to the freshwater input by tributary streams. In this paper, we present the results concerning the monitoring sites shown in Table 3, Fig 1 and S1 Fig, referring to further bibliography for more detailed information (e.g., [31] for CSFOs at VR; [30] for CSFOs at SR; [28] for CSFOs at CR; [32] for CSFO at MR). Except for fish sampling, authorized by the local authorities as requested, no further specific permission was necessary to performing the field activity presented in this study.

**Table 3:** Main characteristics of the monitoring reaches.

Reservoir ID	Site ID	Geographic coordinates	L km	Z m_ASL_	A_NAT_ km^2^	Q_NAT_ m^3^ s^-1^	MF m^3^ s^-1^	S -
**CR**	**C1**	46°29’10.77”N 10°21’27.48”E	6.7	1,216	98	2.5	0.15–0.25	0.034
	**C2**	46°21’11.38”N 10°21’28.39”E	22.9	940	599	15.0	0.9–1.5	0.025
**VR**	**V1**	46°17’20.15”N 10°15’37.28”E	6.0	630	145	2.2	0.24–0.41	0.043
**SR**	**S1**	46°12’10.80”N 10°08’58.65”E	5.6	410	921	24.5	1.65–2.43	0.010
**MR**	**M1**	46°25’45.87”N 9°21’06.99”E	0.2	1,500	25	1.3	0.10	0.123
	**M2**	46°23’51.21”N 9°21’00”.49E	5.1	1,057	124	6.4	0.39	0.017

L = distance from dam, Z = elevation, A_NAT_ = natural catchment area, Q_NAT_ = mean annual natural flow, MF = minimum flow set by law, S = mean slope.

As expected in the study area, the monitored reaches were relatively steep (slope in the range 0.01–0.1, Table 3), and had rather coarse riverbed substrates, mainly composed of boulders, cobbles and pebbles in varying percentages. Channel morphology was step-pool at V1 and M1, and riffle at the remaining sites.

All the investigated channels displayed substantial morphological alterations (i.e., armored streambed due to fine-sediment trapping behind dam structures, embankments, and grade control structures). Moreover, streamflow (hereafter Q) was highly regulated due to the massive hydropower exploitation: in the study area, a mandatory minimum flow was released below intake facilities, ranging from 5 to 10% of the mean annual natural flow (Q_NAT_) estimated at the intake section (Table 3). The baseflow at our monitoring reaches was therefore the specific minimum flow; additional contributions were due to the residual unexploited catchment, and possible upstream spills during high-flows [20].

### Physical monitoring

Q and SSC were continuously gauged in the course of the CSFOs. As previously anticipated, SSC was measured by optical turbidimeters and water samples were collected to perform a-posteriori calibration of the raw turbidity data. Additional samples of turbid water were collected during the CSFOs to measure the grain size of the suspended load, following the procedure described by Espa et al. [31]. Sediment mass flowed in suspension was computed by time integration of SSC multiplied by Q. The sediment mass was then divided by density, estimated according to Yang [33], to quantify the volume of sediment removed during the CSFOs. At MR, the flushed sediment volume was determined by comparing before/after bathymetry [34].

During some CSFOs (i.e., in 2006 and 2007 at VR, and in 2010 at MR) DO was continuously measured by oxygen meter probes at the SSC gauging stations. Analogous spot measurements were carried out during the further CSFOs.

Some CSFOs (i.e., VR in 2008, MR in 2010, and CR in 2011) were also surveyed for riverbed alteration, i.e., increase in fine sediment content of the uppermost riverbed substrate. In-channel sediment deposition was assessed by before/after measurement in selected transects, adopting different techniques (visual estimate, resuspension, McNeil sampling, and measure of thickness by graduated rods) [35].

### Biomonitoring

Trout monitoring was performed at V1, C2 and M2 (Table 3). Quantitative sampling was carried out by electrofishing (removal method with two passes), one time before and one time after the CSFO [32], being the lag-time between fish sampling and the related CSFO usually in the order of one month. The trout were counted, measured for total length and juveniles were differentiated from adults according to the threshold of 170 mm [28]. Density was calculated by dividing fish number to the sampled area, approximately 0.10–0.15 ha. The density reduction obtained by comparing before/after CSFO sampling was considered as apparent mortality, i.e., possible fish migration occurred during the time interval between the two samplings could not be accounted for. The observed apparent mortality of each age class was then compared to the mortality computed through the Newcombe and Jensen formula [27]. At V1 and M2, fishing is forbidden, while it is allowed in spring and summer at C2. Brown trout juveniles (i.e., Mediterranean stock 50–120 mm) are restocked on summer in the investigated reaches.

Benthic macroinvertebrates sampling was performed within the month before and the month after each CSFO (i.e., pre-flushing and 1^st^ post-flushing samples). Further sampling was carried out with variable time resolution (one to six additional samplings per year), depending on the study reach and the year. Sampling was carried out following the standard protocols for the stream quality assessment by the National normative index (see [31] for additional detail about benthos monitoring at V1, and [30] for the other reaches). In this paper, we present the results concerning the basic metrics representative of the main structural aspects of the benthic macroinvertebrates community, i.e., total density and richness (total number of families).

The relationship between the percentage reduction of the benthos metrics after the CSFOs and the corresponding sediment dose was investigated by Pearson product-moment correlation coefficient. The mentioned percentage reduction was computed by comparing the 1^st^ post-flushing sample to the corresponding pre-flushing one. Moreover, we quantified the sediment dose as the natural logarithm of the product between SSC_AVE_ and ED, according to the definition provided by Newcombe and Jensen [27]. No endangered or protected species were involved in this study.

## Results and discussion

### Downstream perturbation

#### SSC and Q patterns during CSFOs

In spite of the overall similarity already introduced, the reported CSFOs displayed significant quantitative differences (Table 4). Different SSCs were gauged downstream of the flushed reservoirs, with values averaged over the entire CSFO approximately spanning one order of magnitude, e.g., 0.8 to 8 g L^-1^ at S1 in 2009 and at C1 in 2011, respectively. The peak values (hereafter SSC_MAX_) had even more pronounced variability, e.g., from few to around 100 g L^-1^.

**Table 4:** Main results of the physical monitoring of the CSFOs.

Reservoir ID	Year -	ED days	Site ID	Q_AVE_ m^3^ s^-1^	SSC_AVE_ g L^-1^	SSC_MAX_ g L^-1^	M_TOT_ 10^3^ t
**CR**	2010	46	**C1**	1.0	3.5	30.2	14.6
			**C2**	3.4	0.3	3.3	4.6
	2011	53	**C1**	1.3	7.9	68.9	70.9
			**C2**	4.8	0.3	6.1	13.9
	2012	40	**C1**	0.9	5.9	99.2	26.1
			**C2**	3.4	0.8	38.2	12.1
**VR**	2006	13	**V1**	3.2	4.7	50.0	17.0
	2007	12		4.4	3.0	11.9	14.0
	2008	13		4.6	3.5	13.6	18.7
	2009	13		3.5	4.0	48.2	18.0
**SR**	2009	16^a^	**S1**	70	0.8	6.2	74.9
	2010	6^a^		60	0.73	3.6	24.0
**MR**	2010	3	**M2**	10	2.6	16.6	21.9^b^

ED = duration of the event; Q_AVE_ = average streamflow, SSC_AVE_ = average suspended sediment concentration, SSC_MAX_ = maximum suspended sediment concentration, M_TOT_ = total flowed mass.

^a^ CSFOs at SR were carried out along non-consecutive days: SSC_AVE_ was computed by time-averaging SSC detected during the flushing days only

^b^ value detected through before/after CSFO bathymetry

The grain size of the flushed sediment was almost entirely below 2 mm. However, the sand content (grain size between 62.5 μm and 2 mm) varied from negligible at VR and CR, where most of the evacuated sediment was silt [28, 31], to approximately 75% at MR [34]. Additional data concerning the grain-size of the suspended sediment sampled during the CSFOs are provided in S1 Table. ED varied more than one order of magnitude, i.e., between few days to approximately two months. Finally, the Q increase during the CSFOs was different at the various investigated reaches. Specifically, the average Q during the flushing (hereafter Q_AVE_) varied from 30%-50% of Q_NAT_ (at C1 and C2, respectively) and rose around 250% of Q_NAT_ at S1.

**Fig 2:**
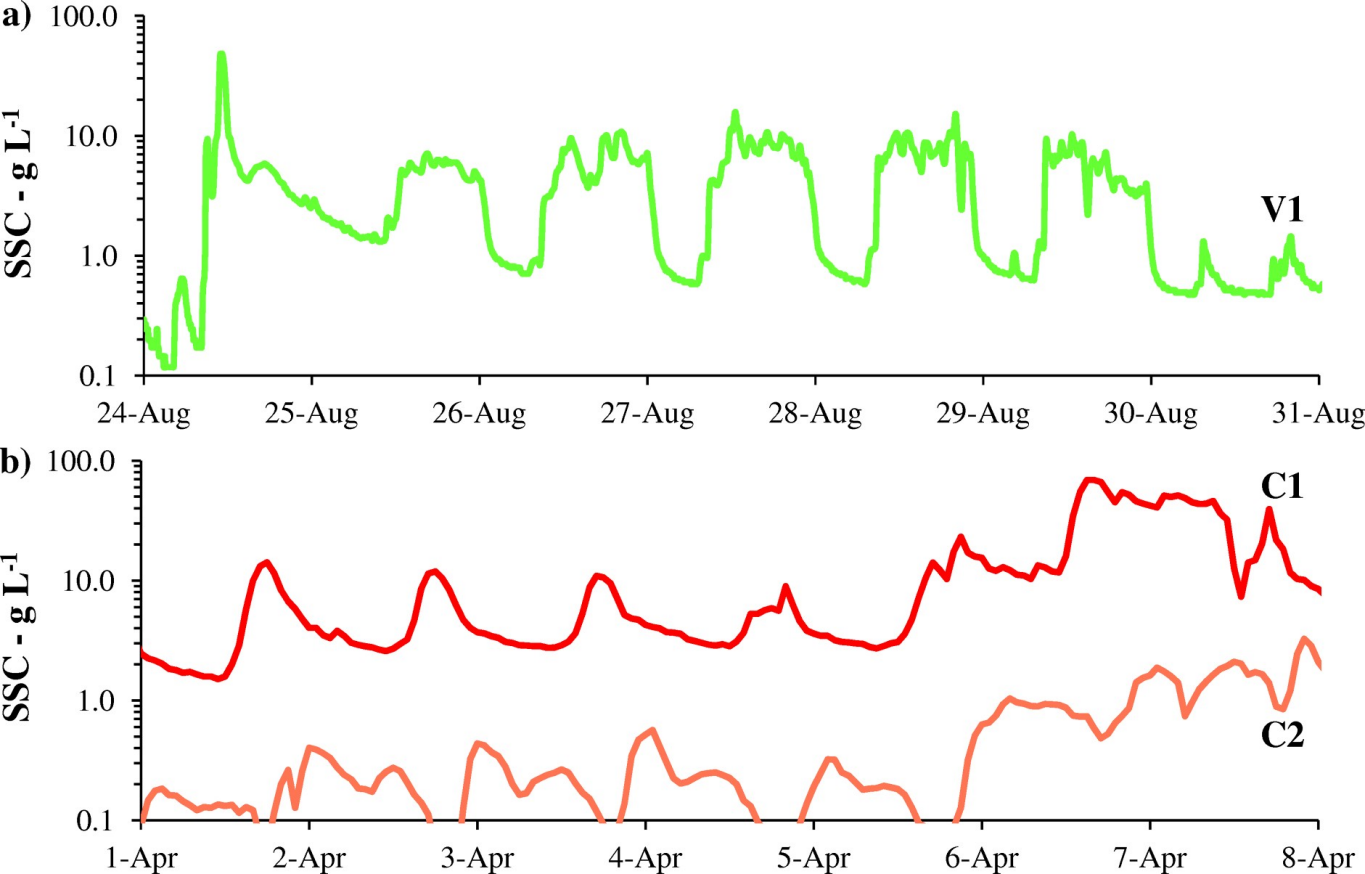
Examples of SSC time-series during the CSFOs. a) SSC detected at V1 during the first week of the 13-days CSFO at VR in 2009. The bottom outlet of the dam was opened on the first day of the CSFO (24-Aug), and SSC peaked around 50 g L^-1^; in the following days, the system was under control, and regular SSC pulses were gauged downstream. b) SSC detected at C1 (dark red) and C2 (light red) during the seventh week of the 53-days CSFO at CR in 2011. The bottom outlet of the dam was opened on 6-Apr, after 47 days of work, and SSC peaked around 70 g L^-1^; in the previous days, sediment was by-passed through the dam and the system was under control, with downstream gauged SSC signals characterized by regular pulses.

Nevertheless, SSC_AVE_ satisfactorily complied the adopted thresholds, as shown in Tables 2 and 4, the only exception being the first CSFO at VR. This compliance was mainly achieved by keeping daily averaged SSC below the adopted threshold, which was feasible when the systems were fully under control. Two examples are provided in Fig 2. Fig 2A concerns the SSC time-series at V1 during the 2009 CSFO at VR: excluding the first day of the event, SSC typically increased up to approximately 10 g L^-1^ during the diurnal dislodging works, and dropped below 1 g L^-1^ over night. SSC detected at C1 and C2 during the 2011 CSFO (Fig 2B) was comparably regular before the bottom outlet of the dam was opened. The SSC time-series of all the reported CSFOs, at least when the related systems were under control, showed similar patterns, characterized by regular daily pulses due to the employment of earth-moving equipment during daytime. A synchronous Q pulse was also operated, particularly at SR and MR.

#### SSC peaks

SSC peaks–even if of short duration–can considerably impair the downstream river environment [36–37], and should be avoided in CSFOs. In general, SSC peaks occur immediately after full draw-down, when riverine flow establishes throughout the emptied reservoir, triggering retrogressive erosion along the flushing channel. Peak SSCs can vary greatly, also when reservoirs are operated in a consistent manner: consequently, related predictions remain uncertain. However, values in the order of 100 g L^-1^ can be anticipated for fine-grained deposits [15]. This was partly confirmed by the reported events, particularly at final emptying of VR in 2006 and 2009 and CR in 2011 and 2012 (Fig 2 and Table 4). In these occasions, SSC control turned out to be particularly challenging and was partly lost. Specifically, difficulties of SSC control at the mentioned stages were also connected to:

- The size of the bottom outlet facilities, i.e., steel pipes of small diameter, not specifically designed for flushing management,
- The degree of siltation of the mentioned facilities, buried in both cases by very thick layers of silt (particularly VR in 2006 and CR in 2011),
- The concurrent and unexpected increase of the water inflow in the emptied reservoir (e.g., CSFO at CR in 2011).

If one only threshold concerning SSC_AVE_ is not considered adequate to support proper downstream protection, multiple thresholds can be adopted. For instance, at SR, where a downstream area of particular interest deserved special attention, the threshold concerning SSC_AVE_ (1.5 g L^-1^) was coupled to a further alert threshold (3 g L^-1^), requiring revision or interruption of the ongoing flushing activity [30]. A multiple thresholds approach was also implemented at the Génissiat Reservoir (Rhône River, France), one of the few reservoirs at global scale where CSFO is performed and documented in the literature [38–39]. There, the SSC threshold for the entire operation (5 g L^-1^, ED around 10 days) is complemented by further restrictions (10 g L^-1^ and 15 g L^-1^ for maximum continuous periods of 6 and 0.5 hours, respectively) [40]. The same protocol was recently adopted to perform a CSFO at the Verbois Reservoir (Switzerland), located along the Rhône River about 40 km upstream from the Génissiat Reservoir [41]. Further advanced schemes to managing siltation by draw-down flushing are reported by Reckendorfer et al. [24], concerning a chain of run-of-the-river hydropower reservoirs located along the upper reach of the Mur River (Austrian Alps). These CSFOs, as supported by large outlet facilities of the dam structures, are carried out only during high flows, and current protocols provide integrated restrictions for SSC, ED, streamflow and season.

Sharp SSC peaking could combine with DO decrease, thus amplifying downstream impacts [37, 42]. SSC and DO are negatively correlated, but relationships between them result intrinsically site-specific [43]. In mountain areas, the extent of DO drops associated with SSC peaks can be mitigated by the generally low water temperatures, intense mixing by turbulence, and low organic content of sediments. Accordingly, we measured DO levels always within the range of baseline condition (i.e., mostly above 10 mg L^-1^ with minimum of 8 mg L^-1^), also during the final emptying of VR during the 2006 CSFO, when SSC peaked up to 50 g L^-1^ (Table 4). Even if our DO records are not complete (for instance, the SSC peak occurred at CR in 2011 –Fig 2B–was not surveyed in terms of DO), we are confident that DO depletion during sediment flushing can be controlled in Alpine contexts by providing adequate dilution flow. Monitoring CSFOs in the Austrian Alps, Reckendorfer et al. [24] obtained comparable results. Similarly, Sumi and Kanazawa [44] reported decreasing DO at SSC peak during the sediment flushing of the Dashidaira Reservoir along the Kurobe River, Japan: however, there, performing flushing during high flows guaranteed acceptable DO levels (minimum DO of 6 mg L^-1^ despite SSC peaking up to 161 g L^-1^ during a flushing in 1999). In different geographic settings, e.g., lowland rivers with higher water temperature and larger organic content of sediment, DO depletion progressing to hypoxia has been documented as a consequence of SSC increase. For instance, Baoligao et al. [45] measured significant DO reduction during the 2009 and 2010 flushing operations through the Xiaolangdi Reservoir along the Yellow River, China. Measured drops were from 8 to 3 and 1.1 mg L^-1^ concurrently to SSC peaks of 65 and 80 g L^-1^, respectively.

#### Riverbed alteration

Riverbed alteration after sediment flushing and its possible control to mitigate downstream environmental impact represent in our opinion a key issue in the future upgrading of CSFOs. In fact, deposition of fine sediment onto formerly coarser riverbed substrates can determine a wide range of effects on the fluvial habitat and possible severe impairment of the river biota [46].

Also from this point of view, our field results are significantly different for the discussed CSFOs.

**Fig 3:**
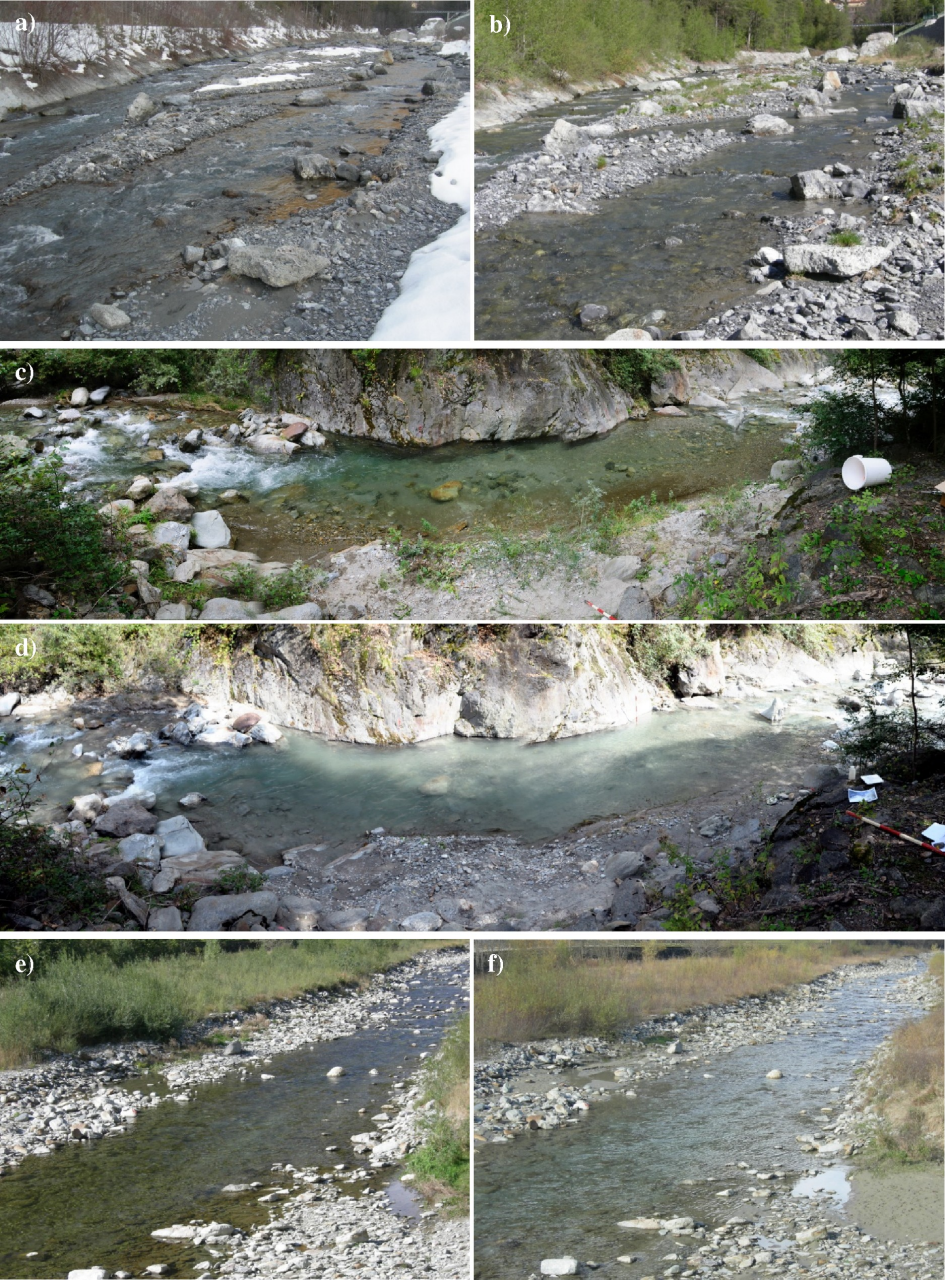
Stream reaches photographed few days before and after CSFOs. Up: Adda River few hundred meters downstream from C1 looking upstream, a) before, and b) after the 2011 CSFO at CR. Middle: pool of the Roasco Stream located some hundred meters upstream from V1, c) before, and d) after the 2008 CSFO at VR (flow is from left to right). Down: Liro Stream at M2 looking upstream, e) before, and f) after the 2010 CSFO at MR.

Macroscopic depositional areas were not detected in case of prevailing silty sediment, as for CSFOs at CR (Fig 3A and 3B) and VR (Fig 3C and 3D). More specifically, by McNeil riverbed sampling, we measured increase in the silt/clay content in the range 1–2.5 kg m^-2^ after the 2008 CSFO at VR [31], and of 0.5–1 kg m^-2^ after the 2011 CSFO at CR [28]. It is worth to remark that the corresponding sediment fluxes were around 19,000 and 71,000 t for the two mentioned events (Table 4). Caution should be exercised in interpreting these quantitative results, due to the low number of transects surveyed (i.e., two and four transects downstream of VR and CR, respectively). Moreover, we restricted our sampling to the riverbed wetted at baseflow condition. However, high efficiency in exporting fine sediment is documented in the literature for mountain river systems, also in extreme sedimentation events [47].

In contrast, after the CSFO at MR, where the flushed sediment was predominantly sand, a more evident riverbed alteration was observed (Fig 3E and 3F). In that case, the amount of the sandy deposits was 300 and 30 kg m^-2^ on average, at M1 and M2, respectively [32]. However, McNeil sampling in the Liro River evidenced a negligible change in the silt/clay content before and after the CSFO, related to the high Q maintained in the course of the event [34].

### Efficiency and cost of CSFOs

**Fig 4:**
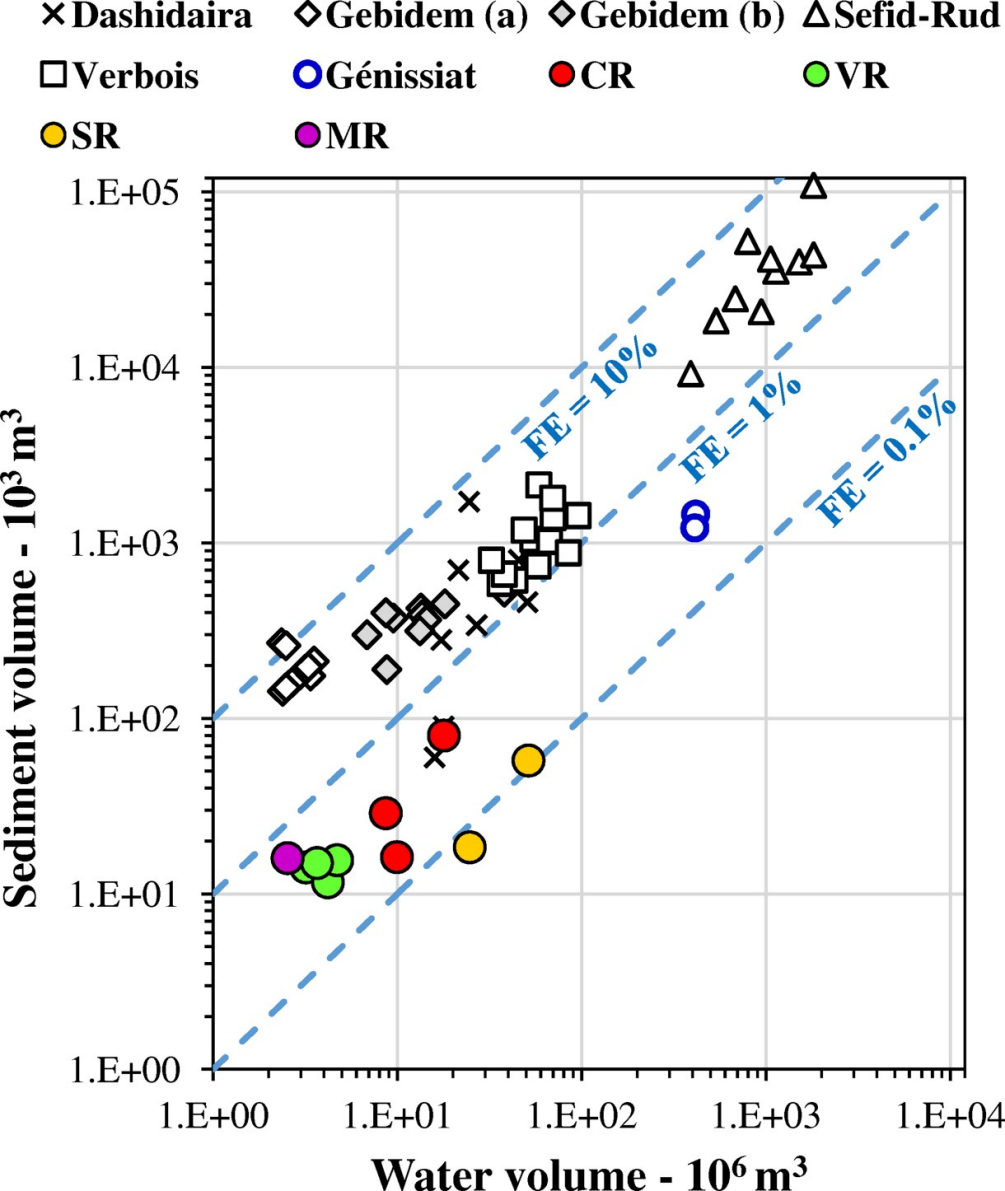
Flushing efficiency (FE) for the reported CSFOs compared to further operations reported in the literature. Sources: Cohen and Briod [48] for thirteen events at Verbois Reservoir, from 1945 to 1981. Morris and Fan [15] for ten events at Sefid-Rud Reservoir, from 1980 to 1990, and for twelve events at Gebidem Reservoir (a), from 1982 to 1993. Sumi and Kanazawa [44] for nine events at Dashidaira Reservoir, from 1995 to 2004. Meile et al. [49] for ten events at Gebidem Reservoir (b), from 2004 to 2013. Peteuil et al. [40] and Guertault et al. [39] for two events at Génissiat Reservoir, in 2003 and 2012.

Flushing efficiency (hereafter FE) can be defined as the ratio between the volume of evacuated sediment and the corresponding volume of water [15]. For our CSFOs, we computed the volume of water by including all the freshwater volumes released during the operations with the specific purpose of mitigating downstream impact. The corresponding efficiency of the ten CSFOs ranged between 0.1 and 0.6% (Fig 4).

Morris and Fan [15] observed that FE can vary widely, and reported prevailing values in the range 2–10%. Moreover, they acutely noted that “A high flushing efficiency is not necessarily synonymous with desirable or effective sediment management … High flushing efficiency may also generate sediment concentrations downstream which are excessive from the standpoint of other users or the environment”.

Comparison with sediment flushing operations reported in the literature (Fig 4) evidences that our CSFOs, as well as the Génissiat one, display the lowest FEs, about one order of magnitude lower than most of the other ones, an evident consequence of the precautionary measures adopted to limit the downstream impact. On the other hand, as discussed in the Supplementary Information (S1 File), FE is only one of the several parameters controlling the downstream impact, at least when predicted on fish according to the model of Newcombe and Jensen [27]: for instance, CSFOs characterized by the same volume of evacuated sediment and the same SEV can have significantly different FE.

The cost for removing the unit volume of sediment ranged from 5 to 45 € m^-3^. These costs include the loss of hydropower and the expenses connected to mechanical excavation. The loss of hydropower is evidently related to the surface elevation of the water volumes utilized for flushing, mostly controlling the potential electricity generation in the downstream hydropower cascade. Accordingly (Table 1), the lowest cost was achieved in the 2009 CSFO at SR, the highest during the 2010 CSFO at CR, where, additionally, the works were long lasting and especially complex. Incidentally, the highest cost is comparable to the maximum value (50 US$ m^-3^) for dry excavation by conventional earth-moving equipment in Los Angeles County reported by Morris and Fan [15].

Even if a comprehensive quantitative evaluation was not performed, it is likely that sediment removal by dredging and transport in a suitable disposal site, both by lorry or by special purpose piping, would have been significantly more expensive. In fact, dredging works inside reservoirs are commonly recognized as particularly expensive [15], and have often limited effectiveness [14]. Moreover, further complexities connected to transport difficulties and scarcity of suitable sites for disposal are especially expected in high mountain environments.

### Biological impacts

The impact of increasing fine sediment load and related streambed alteration on the river biota has been extensively studied, and summarized in several reviews [43, 50–53]. In contrast, only few studies have focused on the biological effects of sediment flushing [12, 24, 36–37, 42, 45, 54–58], thus justifying further research efforts.

From the one hand, contiguous fields of research can support the planning of CSFOs, for example the impact of dam removal [59–61], mining [62], dredging [63], and sediment by-pass tunnel operation [64–65]. However, quantitative guidance, and particularly operational tools to predict the flushing impact require substantial upgrading [6]. A notable exception is the already introduced model by Newcombe and Jensen [27], recently used to predict the flushing impact on downstream fish also in numerical modeling studies [66–67]. Analogous tools for predicting the impact of sediment releases on benthic macroinvertebrates are currently unavailable, at least to our knowledge.

#### Effects on trout

**Fig 5:**
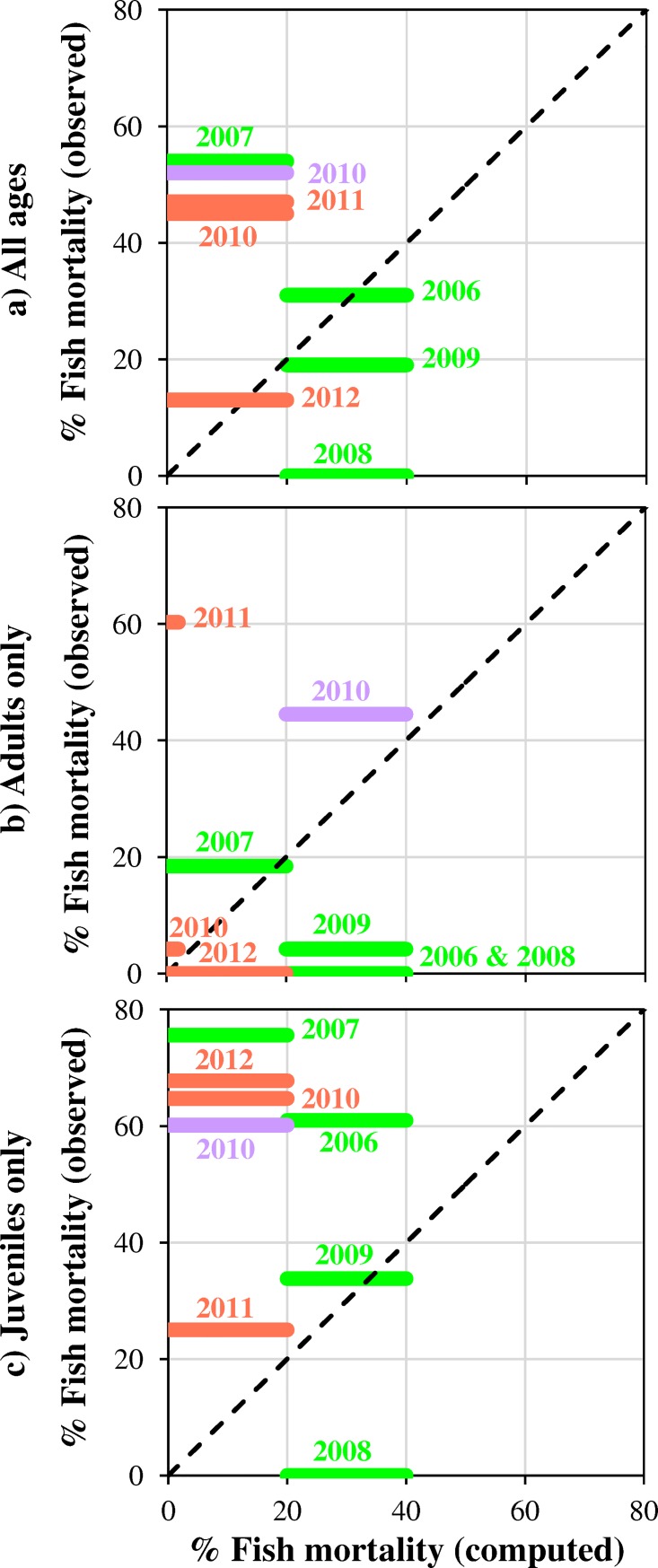
Impact of the CSFOs on trout, and comparison between measured apparent mortality and computed fish mortality. Computation was carried out by data reported in Table 4, according to the model by Newcombe and Jensen [27], and adopting the coefficients reported in S1 File for salmonids: all ages (a), adults only (b), juveniles only (c). The SEVs predicted by the model ranged between 9 and 11, implying 0, 0–20, 20–40% fish mortality, respectively. SEV = 9 was obtained only in case (b), for 2010 and 2011 CSFOs at CR. Trout monitoring was carried out by electrofishing at V1 (green), C2 (light red), and M2 (light purple). Different CSFOs years are reported on the pictures.

The total density of trout decreased after almost all the CSFOs, with largest reductions of approximately 50% (Fig 5). Moreover, a selective pressure on juveniles was demonstrated by their response, significantly different in comparison to adults (Fig 5). The more accentuated sensitivity to sediment pressure at early life stages, well established in the literature [50, 52], was recently documented also for dredging activities [63].

Overall, our trout surveys indicated that the model by Newcombe and Jensen [27] can support the CSFO planning, providing a preliminary estimate of the impact on fish. On the other hand, the heavier impact on juveniles detected in the field was underestimated by the model (Fig 5). Further differences between predicted and observed values (i.e., at C2 after the 2011 event, Fig 5) can be ascribed to the possible bias due to game fishing rather than to underestimation of the model. An analogous tendency of the SEV model to underestimating the acute effects of suspended sediment on carp (*Cyprinus carpio* L.) was noticed by Xu et al. [58] who proposed a revision of the model to improve fitting of experimental data recorded during sediment flushing at Xiaolangdi Reservoir (Yellow River—China). In contrast, Grimardias et al. [57] assessed an overall good performance of the SEV model, reporting that the reduction in fish density observed after the drawdown flushing of the Verbois Reservoir (Rhône River, Switzerland) was consistent with the predictions of this model, for both salmonid and non-salmonid species.

Additional longer-term impacts on trout due to sediment deposition and reduced availability of trophic resources, mainly constituted by invertebrates (see following sections concerning the effects on benthos), can be expected after the CSFOs. In fact, a recent study by Ramezani et al. [68] provided field evidence that increased deposition of fine sediment, as low as 0.1 kg m^-2^, can affect brown trout density and condition. However, these authors [68] did not find a reduction in the prey biomass eaten by trout under sediment pressure, despite this pressure adversely affected food availability. This was probably the consequence of a lower competition for food connected to reduced trout density, or to a lower ability of the prey to find refugees following riverbed alteration by increased fine sediment content. Unfortunately, in our monitored reaches, bias connected to game fishing and restocking prevents evaluating these further potential impacts.

#### Short-term effects on benthos

The short-term impact refers to the contraction of the benthic community in terms of density and richness immediately after the CSFOs. This contraction is related to the physical disturbance due to the SSC increase (i.e., abrasion by transported particles) combined to that related to the flow increase (i.e., increased shear stress and riverbed overturn) and the sediment accumulation (i.e., rapid burial by sediments). Specifically, detectable impacts of increasing SSC on benthos can occur at values considerably lower (i.e., one to two orders of magnitude) than those recorded during the reported CSFOs [62, 69]. Moreover, sediment deposition around few kg m^-2^ or less was reported to determine measurable contraction of benthic assemblages [68, 70].

**Fig 6:**
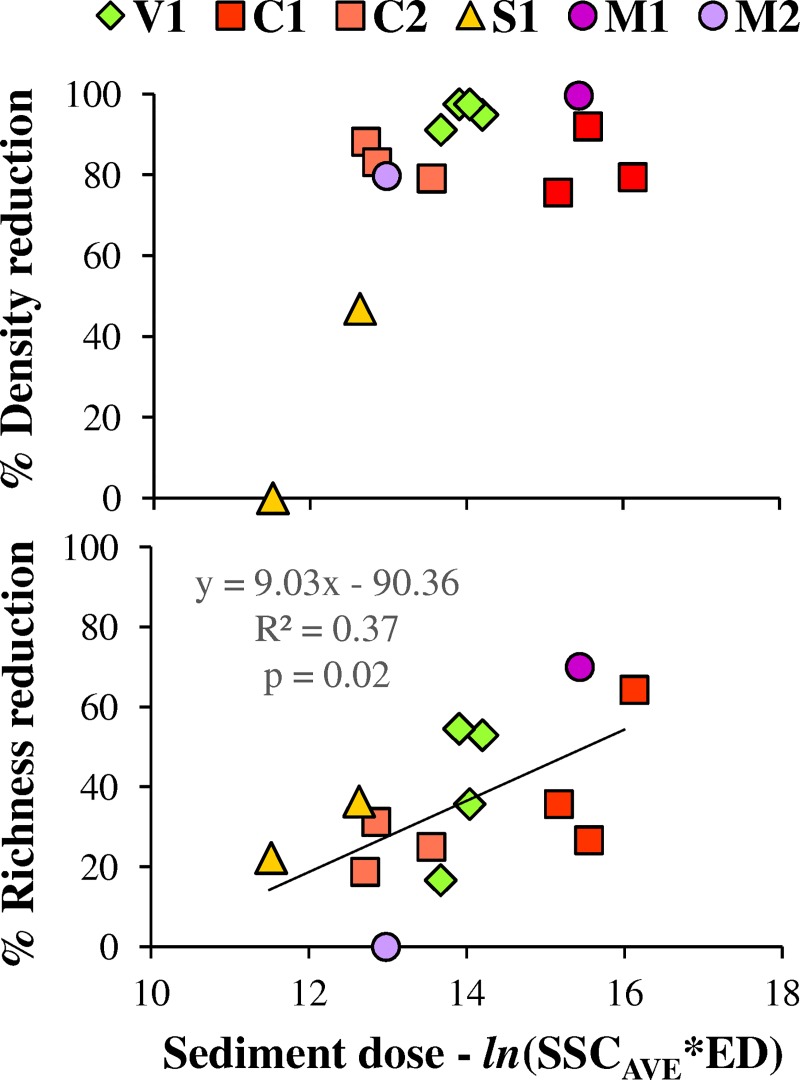
**Benthos contraction in terms of density (*up*) and richness (*down*) short after the CSFOs as a function of sediment dose.** Percentage reduction was computed by comparing the first post-CSFO sample to the corresponding pre-CSFO one. In accordance to Newcombe and Jensen [27], the sediment dose was computed as the natural logarithm of the product between SSC_AVE_ (mg L^-1^) and ED (hours).

As expected, the comparison between samples collected before/after the CSFO evidenced a relevant short-term impact (Fig 6): the density generally decreased more than 70%, and the richness dropped by 10–60%. Similar responses in terms of density reduction to different sediment dose might suggest an asymptotic behavior, i.e., comparable responses of the benthic assemblage once a certain threshold is exceeded: in accordance to the previously quoted literature, it is likely that this threshold is lower than the investigated sediment-doses. The smaller density reduction was detected after the 2010 CSFO at SR: in that case, the sediment dose was lower than for the other CSFOs, but, at the same time, the pre-CSFO sample was already rather poor, due to a further perturbation characterizing the monitoring site (i.e., flow fluctuations due to upstream spills).

The correlation of richness reduction to the sediment dose indicates a taxon-specific response to perturbation induced by the CSFO, according to the different sensitivity to sediment disturbance shown by the various taxa [62, 71–73]. Overall, the linear regression reported in Fig 6 can be adopted as a first-approximation predictive model, supporting the planning of analogous CSFOs. Specifically, the sediment doses causing 0% and 50% richness reduction and determined from this regression are 10 and 15.5, respectively.

Comparable reductions of benthos density and richness were found after sediment by-pass tunnel operations in the Albula River (Switzerland); during these operations sediment volumes of 10^4^−10^5^ m^3^ were by-passed through a reservoir in approximately 10 hours during natural high-flow events [64].

#### Recovery patterns of benthos

**Fig 7:**
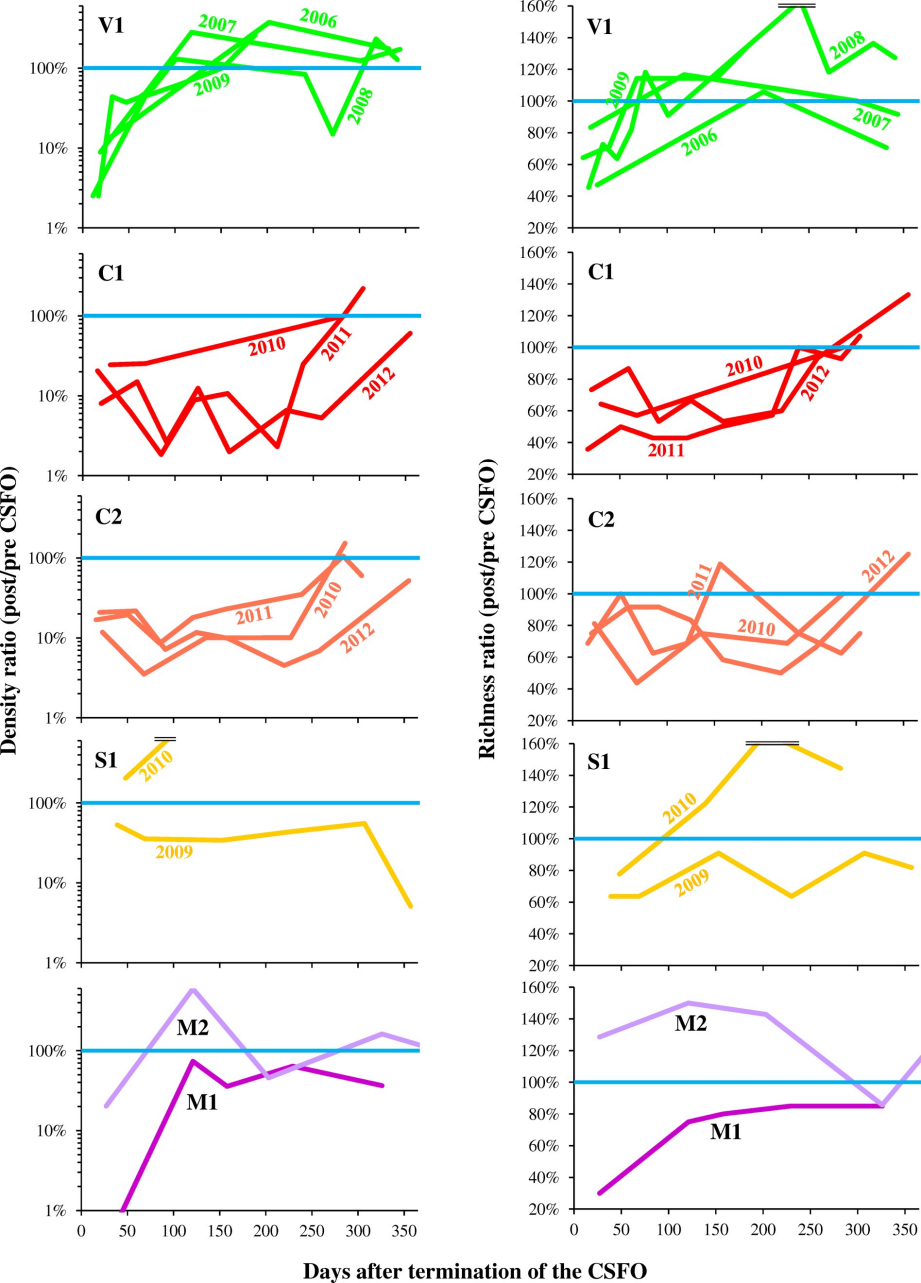
**Temporal evolution of density (*left*) and richness (*right*) of the benthic communities after the CSFOs.** Both density and richness are expressed as the ratio of the current value to the corresponding value detected in the pre-CSFO sampling.

The recolonization trajectories, in general, and more specifically the time employed by the benthic communities to re-gain previous density and richness after the investigated CSFOs displayed different patterns (Fig 7). For example, at V1, where the CSFOs were carried out at the end of the summer (Table 2), sediment deposition was relatively low (as specified above), and a major tributary likely acted as a source of invertebrates, the fastest recovery was recorded (i.e., ca. 3 months after the CSFOs). In contrast, benthos recovery at C1 occurred approximately after 250–300 days, probably as a consequence of the onset of larger seasonal flow in spring, i.e., short after the CSFOs at CR (Table 2), thus adding further disturbance to the benthic community. At both M1 and S1 (after the 2009 CSFO) neither density nor richness recovered to the pre-flushing values within one year after the CSFOs. In fact, as previously remarked, at M1 noticeable sand deposition was detected after the CSFO. At S1 the Q fluctuation was identified as the driving factor shaping the benthic community and confounding the detection of the actual CSFO effects [30]. Indeed, after the 2010 CSFO, both density and richness increased. Faster recovery was observed at sites M2 and C2 in comparison to the corresponding upstream sites M1 and C1, thus demonstrating the effectiveness of the adopted mitigation measures, particularly the freshwater release through regulated tributaries. Consistency between the recovery patterns after the different CSFOs carried out in consecutive years at VR and CR was noticed, and no cumulative effects, i.e., a progressive decline of the benthic community in the course of the years, were detected.

Overall, the benthos recolonization appears controlled by a complex mix of factors, rather than by the sediment dose only. These factors include the flushing season [72] and the persistence of the riverbed alteration [32, 37], as well as site specificities as macroinvertebrate supply by undisturbed tributaries and further anthropogenic disturbance other than CSFO.

Recovery times in the order of few months were also detected after sediment by-pass tunnel operations [64], while a longer time was reported for small dam removal, i.e., 15–20 months [61]. Comparably long recovery time (i.e., around 2 years) was also documented after a sediment flushing event in the western Italian Alps [37]. However, in that case, sediment evacuation was carried out due to a safety-related emergency (i.e., not a controlled operation); moreover, noticeable deposition of fine sediment affected the investigated stream reaches, at least unless artificial floods were released to rework the streambed substrate.

### Management considerations

In general, managing reservoirs siltation through CSFOs implies a complex assessment of interrelated technical, economical, and environmental issues, only schematically outlined in this paper, and deserving in our opinion extensive research effort to be comprehensively approached.

A basic issue concerns the FE, previously defined as the ratio between the volumes of evacuated sediment and of freshwater employed for flushing. In our investigation of Alpine hydropower reservoirs, FE resulted significantly lower in comparison to not-controlled events reported in the literature, up to more than one order of magnitude. However, the impact detected downstream was not negligible, thus implying further potential loss of regulated water and hydropower, and ultimately higher and even intolerable cost, in view of additional refinement of the CSFOs. We think that the search for optimized solutions, probably involving combined sediment management strategies [6, 13], requires further scientific deepening. This issue could assume special interest for reservoirs located in the European Alps, where the temperature increase expected in the next decades is particularly intense [74], and the related glacier retreating and change in precipitation pattern are foreseen to affect hydropower [75], as well as sediment export from formerly glacier-covered catchments [76].

Further management issues are specifically related to the practical implementation of CSFOs. For instance, the alternative between smaller, more frequent flushing operations as opposed to larger but more sporadic ones is still under debate [57], particularly if sediment flushing does not represent an occasional event [37], but is adopted as a recursive sediment management technique. Analogous uncertainty concerns the detailed flushing schedule, i.e., whether alternating sediment pulses and clearer water releases during a CSFO day and/or alternating flushing and non-flushing days within the same CSFO (e.g., this study) is more advisable than evacuating at constant sediment concentration, at least from the standpoint of reducing the downstream impact. Finally, mitigating the downstream effects after CSFOs by carefully managing the streamflow in regulated river reaches has received so far only limited attention, mostly concentrated on artificial flooding [37], rather than specifically focusing on the improvement of the environmental flows [20].

Due to its simple structure, the model by Newcombe and Jensen [27], here adopted for preliminarily predicting the CSFOs effects on trout, might be questioned when significant temporal variation of SSC occurs during the CSFO, particularly in case of uncontrolled peaks, but also for controlled pulses. In this regards, the improvement of the model or the development of more advanced modeling tools appear necessary to support further progress in the CSFO practice. Nevertheless, our electrofishing data indicated that the model of Newcombe and Jensen provided reasonable estimates, although a tendency to underestimate trout mortality was noticed, particularly concerning the younger specimens. The higher sensitivity of the juveniles to sediment pressure confirms that when trout are most vulnerable, i.e., during the egg to young fry stages, CSFOs should be generally avoided. This agrees with the general concept that, as already pointed out by several authors [13, 15], the high-flow season should be preferred for flushing sediment, thus mimicking the natural occurrence of increased sediment load, as naturally experienced by the river biota. This objective is achieved to a considerable extent by adopting advanced CSFO protocols [24] and special-purpose structures, like sediment by-pass tunnels [64]. However, technical reasons can make flushing during high-flows difficult, if not unfeasible. In our examples, this was particularly true at CR and VR, due to low-capacity bottom outlets structures, completely unsuitable for flushing during high-flows [28, 31]. In these cases, removal by electrofishing and repopulation before/after the CSFO could be considered as suitable mitigation measures, possibly operating repopulation campaigns after a certain recovery of both the riverbed and the benthos had occurred. In this respect, we noticed that significant contraction of the macroinvertebrate assemblages in terms of density can be expected short after the flushing, roughly averaging 80% in the investigated CSFOs, and irrespectively of the sediment dose (here defined as the natural logarithm of duration multiplied by sediment concentration). Moreover, we observed that in the mid-term, macrobenthos recovery generally occurred, but the time employed to recover pre-flushing standard and the related trajectories appeared mostly related to the flushing season and to site specificities rather than to the sediment dose. This finding is consistent with the central role played by the functional connectivity with tributaries, already evidenced by further authors, and potentially providing refuge for fish [57] and source of recolonizing macroinvertebrates [77, 78], and which may significantly increase the biota resilience to the CSFO impacts.

## Conclusions

Sediment flushing can tackle reservoir siltation and improve sediment flux through regulated rivers, which commonly result sediment depleted due to trapping behind dam structures. However, as pointed out since the 1980s, sediment flushing can be highly detrimental for downstream environment and instream structures. To limit these drawbacks, sediment flushing through reservoirs can be controlled, but CSFOs are only marginally discussed in the literature. In this regard, the field investigation carried out to assess the environmental impact of the CSFOs discussed in this paper provides a dataset of great practical significance. In fact, these CSFOs displayed significant quantitative differences in terms of evacuated mass, downstream sediment concentration and water flow (both peak and averaged values), grain-size, duration and season of the works.

More extensive field evidence and advanced modeling tools are required in our opinion to improve quantitative prediction of the downstream effects of CSFOs. This can support the planning stage of the projects and their integration in a comprehensive sediment management strategy, aimed to sustain long-term utilization of storage, improving at the same time the environmental quality of impounded rivers.

## Supporting information

S1 Fig Elevation profile of the investigated rivers. Sketch of the hydropower cascades (R. = reservoir; HPP = hydropower plant) and position of the monitoring reaches (colored circles) are shown. a) Adda and Roasco. b) Liro and Scalcoggia.
(TIF)

S2 Fig Valgrosina Reservoir during the 2008 CSFO. Pictures are taken from the dam crest. a) Immediately after full draw-down. b) At the end of the CSFO.
(TIF)

S3 Fig Streamflow increase in the Adda River during the 2011 CSFO at CR. The grade-control structure shown in the pictures is located 14.2 km downstream from the Cancano Dam, roughly in the middle of the stretch between C1 and C2. Pictures are taken from the western bank, looking upstream. a) Before the CSFO. b) During the CSFO. c) After the CSFO.
(TIF)

S1 File CSFO parameters and effects on fish at planning stage. (PDF)

S2 File Raw data of trout and benthic macroinvertebrates. In this file, one spreadsheet (“trout data”) concerns fish data plotted in Fig 5, one spreadsheet (“benthic macroinvertebrates data”) concerns benthic macroinvertebrates data plotted in Figs 6 and 7.
(XLSX)

S1 Table Grain-size of the sediment flushed during some CSFOs. (PDF)
