# Supplementary figures and images for: Tackling reservoir siltation by controlled sediment flushing: Impact on downstream fauna and related management issues
Source: PLoS One. 2019 Jun 24;14(6):e0218822. doi: 10.1371/journal.pone.0218822 (PMC6590828; doi:10.1371/journal.pone.0218822)

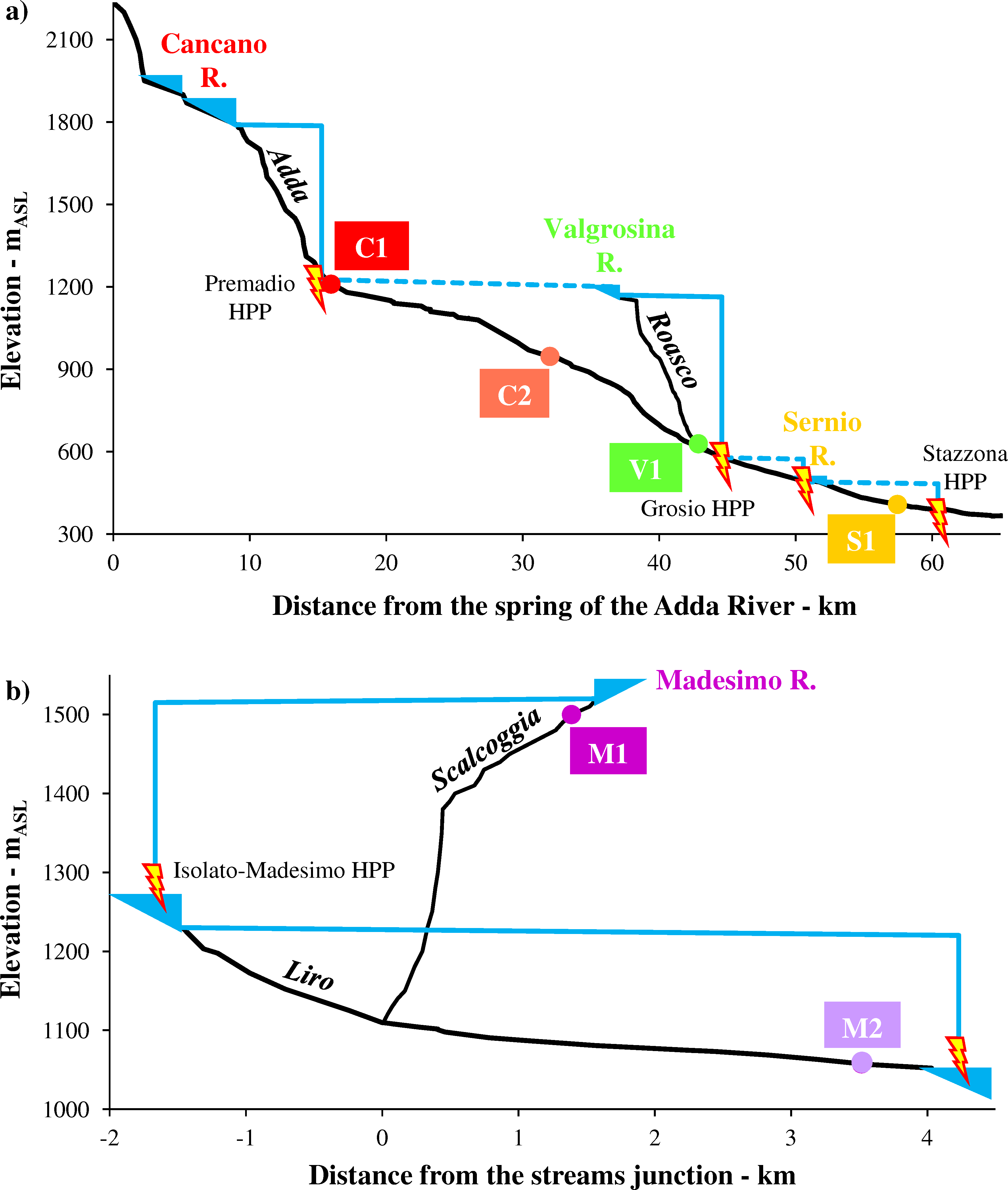

Supplement: S1 Fig — Sketch of the hydropower cascades (R. = reservoir; HPP = hydropower plant) and position of the monitoring reaches (colored circles) are shown. a) Adda and Roasco. b) Liro and Scalcoggia. (TIF) [file pone.0218822.s001.tif]

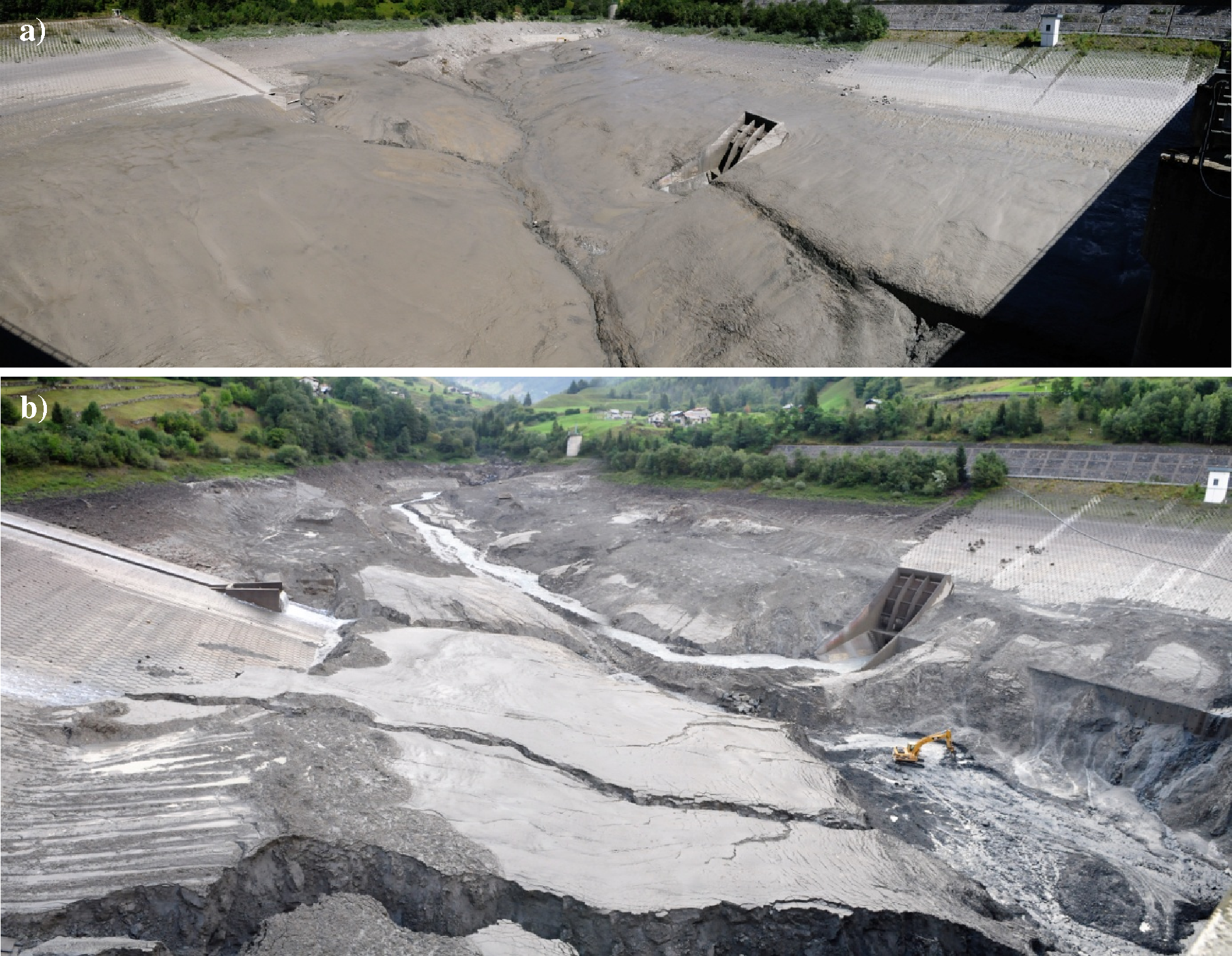

Supplement: S2 Fig — Pictures are taken from the dam crest. a) Immediately after full draw-down. b) At the end of the CSFO. (TIF) [file pone.0218822.s002.tif]

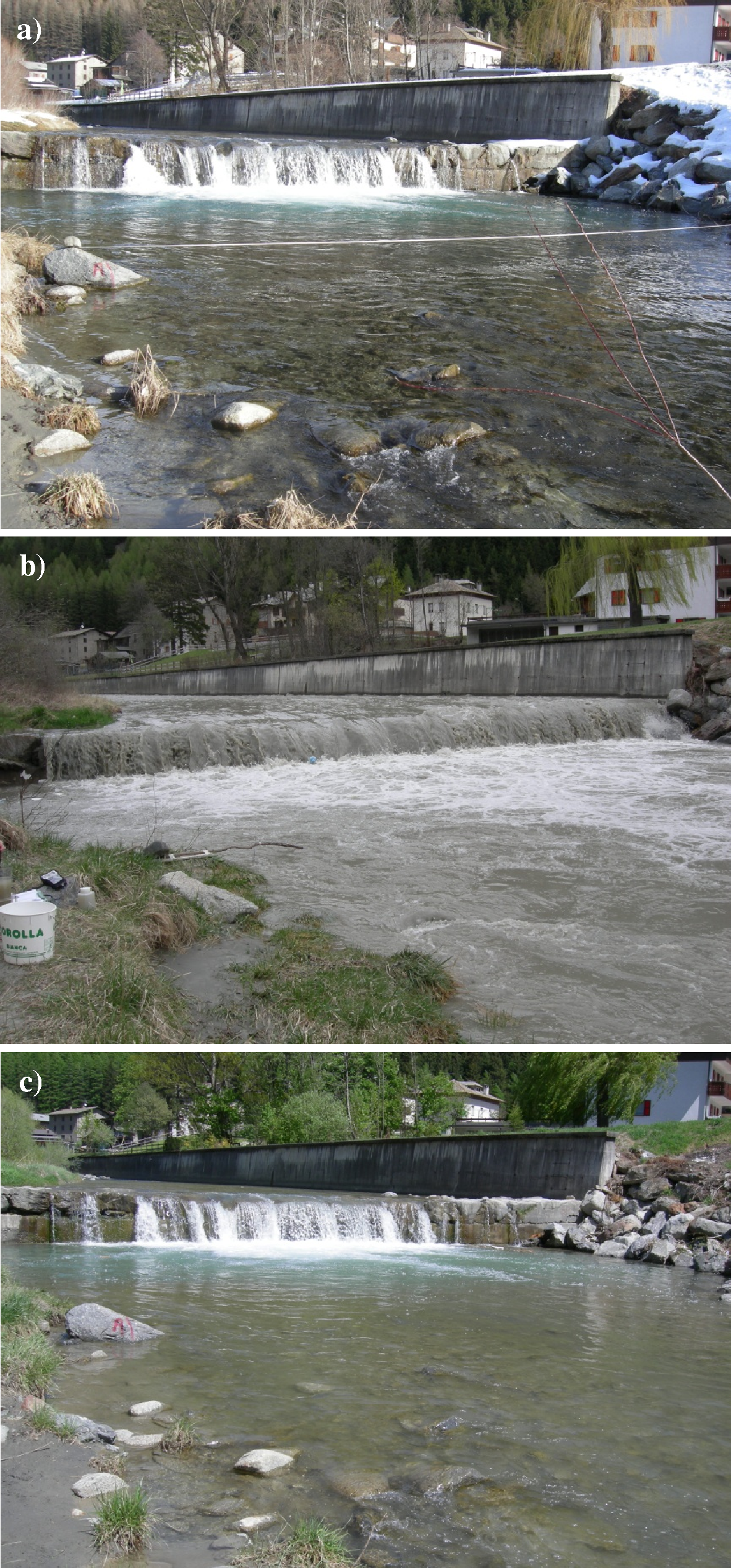

Supplement: S3 Fig — The grade-control structure shown in the pictures is located 14.2 km downstream from the Cancano Dam, roughly in the middle of the stretch between C1 and C2. Pictures are taken from the western bank, looking upstream. a) Before the CSFO. b) During the CSFO. c) After the CSFO. (TIF) [file pone.0218822.s003.tif]
