# Supplementary material for: Tackling reservoir siltation by controlled sediment flushing: Impact on downstream fauna and related management issues
Source: PLoS One. 2019 Jun 24;14(6):e0218822. doi: 10.1371/journal.pone.0218822 (PMC6590828; doi:10.1371/journal.pone.0218822)
Supplement: S1 File — (PDF) [file pone.0218822.s004.pdf]

CSFO parameters and effects on fish at planning stage

When planning a CSFO the following essential parameters should be determined:

- Volume of sediment to be flushed (V_S – m^3)
- Corresponding mass of sediment (M_S – t)
- Duration of the event (ED – days)
- Volume of water for transporting and diluting sediment downstream (V_W – m^3)
- Corresponding streamflow averaged over the ED (Q_{AVE} – $m^3\ s^{-1}$)
- Suspended sediment concentration averaged over the ED (SSC_{AVE} – $g\ L^{-1}$)
- Flushing efficiency (FE)
- Severity of ill effect on fish (SEV), according to the Newcombe and Jensen [27] formula reported below (eq 1)

$$SEV = A + B\times\ln(ED) + C\times\ln(SSC_{AVE}) \qquad (1)$$

Where:
ED is in hours and SSC_{AVE} is in mg L^{-1}, and
A = 1.0642; B = 0.6068; C = 0.7384 (salmonids – all ages)
A = 1.6814; B = 0.4769; C = 0.7565 (salmonids – adults only)
A = 0.7262; B = 0.7034; C = 0.7144 (salmonids – juveniles only)

Depending on the specific layout under consideration, some parameters are established *a-priori*, and the remaining are computed according to available equations/formulas.

In the following, we show one possible algorithm and summarize the main outcomes. In order to provide a quantitative example, we adopt scales roughly comparable to those of the CSFO at VR.

a. V_S is fixed first, representing the main goal of the CSFO
b. M_S is determined immediately, adopting a suitable value of the bulk density of sediment (ρ_S – kg m^{-3})
c. SEV is fixed first as well, providing the accepted level of impact on downstream fish community
d. If another variable is assumed (V_W, Q_{AVE}, SSC_{AVE}, ED, FE), then the remaining four are determined univocally by basic algebra as shown below

In regulated systems, it is highly probable that Q_{AVE} or V_W, quantifying the water available to perform the CSFO, are the main candidate variables to be fixed at step d. An example is reported below.

a. V_S = 15,000 m^3
b. M_S = 18,750 t (i.e., ρ_S = 1,250 kg m^{-3})
c. Three SEVs are considered, 9, 10, and 11
d. Q_{AVE} is varied between two orders of magnitude (1-100 $m^3\ s^{-1}$), keeping in mind that the actual value is around few cubic meters per second (say 5 $m^3\ s^{-1}$)

When M_S, SEV, and Q_{AVE} are fixed, eq 1 can be solved univocally for ED; in fact, $SSC_{AVE} = M_S\ (Q_{AVE}\times ED)^{-1}$. Once ED is determined, $V_W = Q_{AVE}\times ED$, $SSC_{AVE} = M_S\ V_W^{-1}$, and $EF = V_S\ V_W^{-1}$

are immediately obtained. Results are plotted in Fig 1, where constant SEV lines are plotted in different colors.

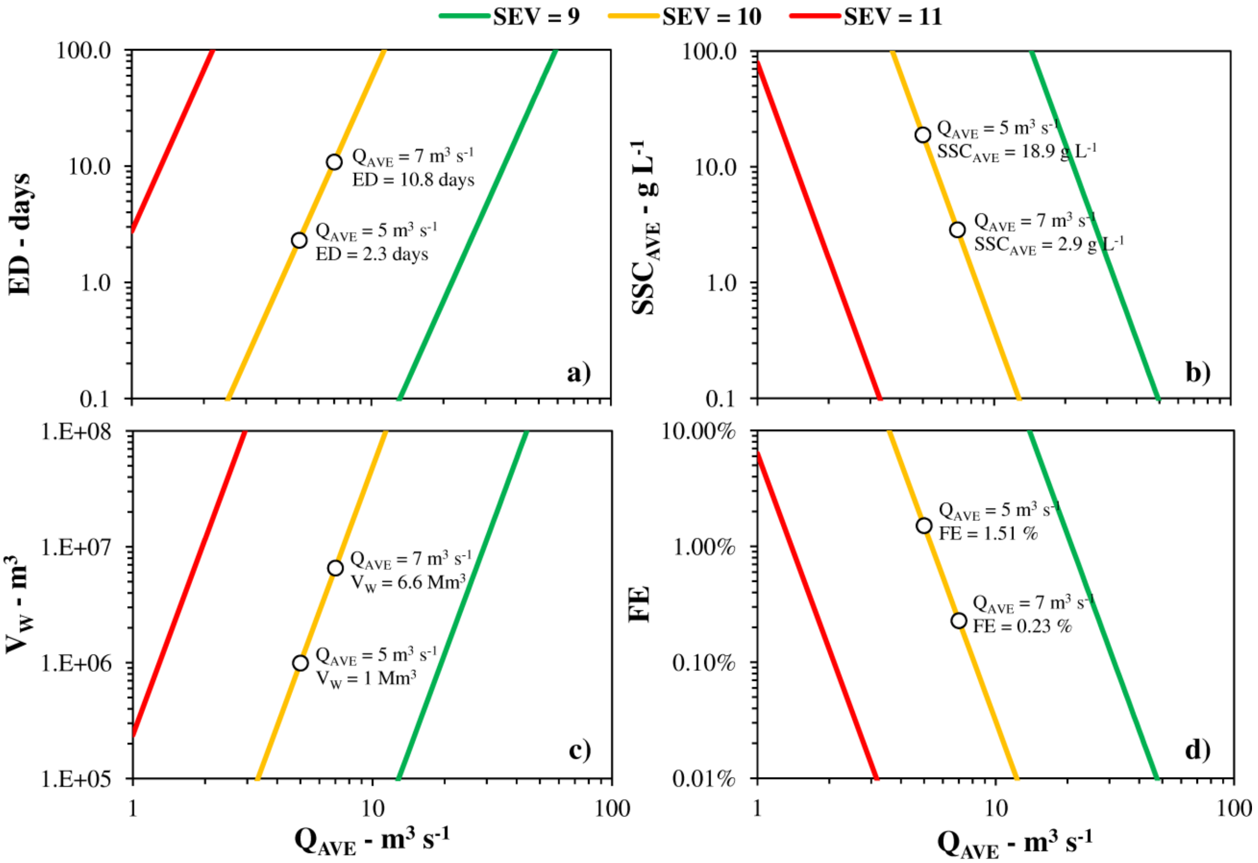

Fig 1. **Basic CSFO parameters as a function of Q_{AVE} for three different values of SEV.** a) ED. b) SSC_{AVE}. c) V_W. d) FE.

It is immediately seen that different SEVs are characterized by clearly distinguished ranges of Q_{AVE}, at least when values plotted on the vertical axes are realistic. This implies that when the Q_{AVE} allowed by the system is quite constrained, the achievable SEV is almost determined.
However, for a given SEV, variations of Q_{AVE} not very large and realistically compatible with the analyzed system can give significant variation of the CSFO parameters. For instance, it is shown that lower Q_{AVE} can give higher FE and shorter ED (which is advantageous in terms of reservoir management), provided that SSC_{AVE} is increased (which can be technically difficult and can give downstream problems in terms of increased deposition and dissolved oxygen - DO - depletion).

If V_W is considered instead of Q_{AVE}, analogous considerations can be done. As for a given V_S fixing V_W is equivalent to fix SSC_{AVE} ($SSC_{AVE} = M_S V_W^{-1}$), we think to improve the clarity of the example by discussing this case in terms of SSC_{AVE}. As previously done, an analogous example is reported below.

a. V_S = 15,000 m^3 (as in previous example)
b. M_S = 18,750 t (i.e., ρ_S = 1,250 kg m^{-3}) – (as in previous example)

c. Three SEVs are considered, 9, 10, and 11 (as in previous example)
d. SSC_{AVE} is varied between two orders of magnitude (0.1-10 g L^{-1}), keeping in mind that the actual value is around few grams per liter

When M_S, SEV, and SSC_{AVE} are fixed, $V_W = M_S\ SSC_{AVE}^{-1}$ and $FE = V_S\ V_W^{-1}$ can be computed immediately. Then, eq 1 can be solved univocally for ED. Finally, $Q_{AVE} = V_W\ ED^{-1}$ can be determined. Results are plotted in Fig 2.

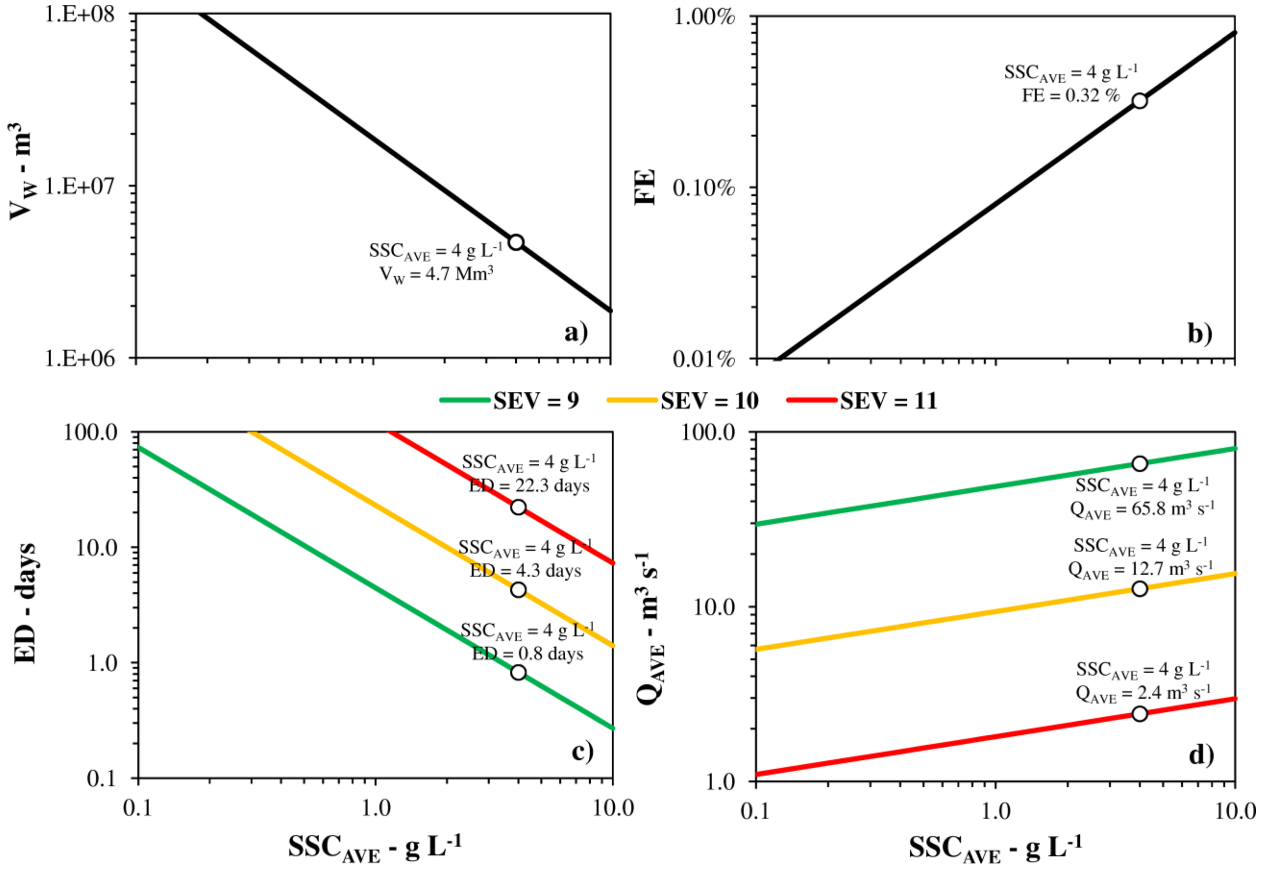

Fig 2. Basic CSFO parameters as a function of SSC_{AVE} for three different values of SEV. a) V_w. b) FE. c) ED. d) Q_{AVE}.

When V_S is fixed, if SSC_{AVE} is decided, then V_W and FE are univocally determined, independently from the adopted SEV. However, different SEVs give significantly different ED. Specifically, the lower the SEV the shorter the ED, looking advantageous from both the reservoir management perspective and the downstream impact. However, the Q_{AVE} required can become unacceptable, also in terms of carrying capacity of the downstream channel.
