# Supplementary material for: Tackling reservoir siltation by controlled sediment flushing: Impact on downstream fauna and related management issues
Source: PLoS One. 2019 Jun 24;14(6):e0218822. doi: 10.1371/journal.pone.0218822 (PMC6590828; doi:10.1371/journal.pone.0218822)
Supplement: S1 Table — (PDF) [file pone.0218822.s006.pdf]

S1 Table. Grain-size of the sediment flushed during some CSFOs. Characteristic diameters of the sediment collected at the monitoring sites during some CSFOs. [a] Sediment was sampled in the emptied reservoir, [b] grain-size analysis was carried out only by dry sieving (44% was in the silt-clay range, i.e., $d < 62.5$ μm)

Reservoir ID	Site ID	Year	d_{16} μm	d_{50} μm	d_{84} μm	References
CR	CR[a]	2010	4.5	16.4	33.3	[28]
VR	V1	2008	5.3	15.8	39.7	[31]
		2009	4.9	13.4	29.9	[31]
SR	S1[b]	2010	n.a.	93.0	392.3	[30]
MR	M2	2010	9.8	32.9	93.8	[34]
